# Predictive Modeling for Perinatal Mortality in Resource-Limited Settings

**DOI:** 10.1001/jamanetworkopen.2020.26750

**Published:** 2020-11-18

**Authors:** Vivek V. Shukla, Barry Eggleston, Namasivayam Ambalavanan, Elizabeth M. McClure, Musaku Mwenechanya, Elwyn Chomba, Carl Bose, Melissa Bauserman, Antoinette Tshefu, Shivaprasad S. Goudar, Richard J. Derman, Ana Garcés, Nancy F. Krebs, Sarah Saleem, Robert L. Goldenberg, Archana Patel, Patricia L. Hibberd, Fabian Esamai, Sherri Bucher, Edward A. Liechty, Marion Koso-Thomas, Waldemar A. Carlo

**Affiliations:** 1 University of Alabama at Birmingham; 2 RTI International, Research Triangle Park, North Carolina; 3 UTH-Children’s Hospital, Lusaka, Zambia; 4 University Teaching Hospital, Lusaka, Zambia; 5 University of North Carolina School of Medicine, Chapel Hill; 6 Kinshasa School of Public Health, Kinshasa, Democratic Republic of Congo; 7 KLE Academy of Higher Education and Research, J. N. Medical College, Belgaum, India; 8 Thomas Jefferson University, Philadelphia, Pennsylvania; 9 INCAP, Guatemala City, Guatemala; 10 University of Colorado, Denver; 11 Aga Khan University, Karachi, Pakistan; 12 Columbia University, New York, New York; 13 Lata Medical Research Foundation, Datta Meghe Institute of Medical Sciences, Nagpur, India; 14 Boston University School of Public Health, Boston, Massachusetts; 15 Moi University School of Medicine, Eldoret, Kenya; 16 Indiana University School of Medicine, Indianapolis; 17 Eunice Kennedy Shriver National Institute of Child Health and Human Development, Bethesda, Maryland

## Abstract

**Importance:**

The overwhelming majority of fetal and neonatal deaths occur in low- and middle-income countries. Fetal and neonatal risk assessment tools may be useful to predict the risk of death.

**Objective:**

To develop risk prediction models for intrapartum stillbirth and neonatal death.

**Design, Setting, and Participants:**

This cohort study used data from the Eunice Kennedy Shriver National Institute of Child Health and Human Development Global Network for Women’s and Children’s Health Research population-based vital registry, including clinical sites in South Asia (India and Pakistan), Africa (Democratic Republic of Congo, Zambia, and Kenya), and Latin America (Guatemala). A total of 502 648 pregnancies were prospectively enrolled in the registry.

**Exposures:**

Risk factors were added sequentially into the data set in 4 scenarios: (1) prenatal, (2) predelivery, (3) delivery and day 1, and (4) postdelivery through day 2.

**Main Outcomes and Measures:**

Data sets were randomly divided into 10 groups of 3 analysis data sets including training (60%), test (20%), and validation (20%). Conventional and advanced machine learning modeling techniques were applied to assess predictive abilities using area under the curve (AUC) for intrapartum stillbirth and neonatal mortality.

**Results:**

All prenatal and predelivery models had predictive accuracy for both intrapartum stillbirth and neonatal mortality with AUC values 0.71 or less. Five of 6 models for neonatal mortality based on delivery/day 1 and postdelivery/day 2 had increased predictive accuracy with AUC values greater than 0.80. Birth weight was the most important predictor for neonatal death in both postdelivery scenarios with independent predictive ability with AUC values of 0.78 and 0.76, respectively. The addition of 4 other top predictors increased AUC to 0.83 and 0.87 for the postdelivery scenarios, respectively.

**Conclusions and Relevance:**

Models based on prenatal or predelivery data had predictive accuracy for intrapartum stillbirths and neonatal mortality of AUC values 0.71 or less. Models that incorporated delivery data had good predictive accuracy for risk of neonatal mortality. Birth weight was the most important predictor for neonatal mortality.

## Key Points

**Question:**

Can prenatal and postdelivery variables accurately predict the risk of stillbirth and neonatal deaths in resource-limited settings of low- and middle-income countries?

**Findings:**

Using advanced machine learning–based modeling techniques on a large multicountry prospective maternal and neonatal database, this cohort study found that the prediction accuracy of models for risk of stillbirth and neonatal death using variables before delivery is low, but the prediction accuracy for neonatal death can be improved by including postdelivery variables. Birth weight was the most important predictor of neonatal mortality.

**Meaning:**

Models that include postdelivery variables have good prediction accuracy for neonatal deaths.

## Teaser

This cohort study evaluates the accuracy of a machine learning–based model to predict risk of stillbirth and neonatal mortality in low- and middle-income countries.

## Introduction

The neonatal period is the period in life with the highest risk for mortality.^1^ Annually, 2.5 million neonatal deaths and 2.6 million stillbirths occur globally, of which 1.3 million are intrapartum stillbirths.^2^ It is estimated that approximately 98% of all neonatal and perinatal deaths occur in low- and middle-income countries.^3,4,5^ However, almost all the published literature on identifying predictors of fetal and neonatal mortality and risk scoring tools are based on data from high-income countries. Data from low- and middle-income countries are limited to small sample size studies that lack validation with an independent sample. Additionally, machine learning prediction models may perform better than conventional models when applied to large data sets given their ability to delineate complex relationships and identify novel interactions between variables.^6,7,8,9,10,11^ Although machine learning–based prediction models are expected to perform better with large data sets, this hypothesis has not been convincingly tested with a good quality prospectively collected population database.^6,7,8,11^

We aimed to develop a risk assessment tool for intrapartum stillbirth and neonatal mortality that would include maternal and neonatal variables from a prospective multicountry maternal and neonatal database. We compared various conventional and advanced machine learning–based, analytical modeling methods at specific time points to establish individual predictive accuracies of the models. We tested the hypothesis that intrapartum stillbirth and neonatal mortality risk prediction models that include antenatal and delivery variables provide a high accuracy. Additionally, we also tested whether advanced machine learning–based models have higher predictive accuracy than a conventional logistic regression model.

## Methods

### Study Design and Participants

The study was conducted in the Eunice Kennedy Shriver National Institute of Child Health and Human Development Global Network for Women’s and Children’s Health Research, which includes clinical sites in resource-limited settings in South Asia (India and Pakistan), Africa (Democratic Republic of Congo, Zambia, and Kenya), and Latin America (Guatemala). A population-based Global Network Maternal Newborn Health Registry (GN-MNHR) vital registry was established in 2009.^12^ Pregnant women and their 502 648 offspring participating in the GN-MNHR database from January 1, 2010, to December 31, 2018, were included. The description of the sites and the cluster settings has been reported.^13^ The GN-MNHR database includes data starting with the initial prenatal visit and up to 42 days after delivery of study participants. The GN-MNHR data processes include close quality monitoring and quality improvement interventions both at local and central levels to ensure data completeness and quality.^12^ The GN-MNHR definitions were used to define variables and outcomes as reported previously.^13^ The GN-MNHR database has been reviewed and approved by all sites’ ethics review committees and the institutional review boards at each US partner university and at the data coordinating center (RTI International). All women provided written informed consent for participation in the GN-MNHR database, including data collection and the follow-up visits. The study is reported as per the Transparent Reporting of a Multivariable Prediction Model for Individual Prognosis or Diagnosis (TRIPOD) reporting guideline for multivariable prediction modeling reporting.^14^ The NICHD Global Network Maternal Newborn Health Registry is registered with ClinicalTrials.gov (NCT01073475).

### Outcomes

The outcome variables were intrapartum stillbirth and neonatal mortality. Intrapartum stillbirth was defined as nonmacerated stillbirth presumably occurring during labor. Neonatal mortality was defined as death up to 28 days after birth. Potential risk factors (variables) for the outcome were selected from the database based on the existing literature and relevancy to the outcomes.

### Statistical Analysis

The risk factors were added sequentially into 4 scenario data sets: (1) prenatal (variables until first prenatal care visit), (2) predelivery (variables until just before delivery), (3) delivery and day 1 (delivery/day 1), and (4) postdelivery through day 2 (postdelivery/day 2). Day 0 was defined as the calendar day of birth. Day 1 and day 2 were defined as the subsequent calendar days. We evaluated mortality outcomes using potential risk factor sets with sequentially additional variables to determine whether additional potential risk factors improved outcome predictive accuracy.^15^ The first 2 scenario models (prenatal and predelivery) evaluated the outcome of intrapartum stillbirth and neonatal mortality. The third scenario model (delivery/day 1) evaluated the outcome of neonatal mortality on days 2 through 27. The fourth scenario model (postdelivery/day 2) evaluated the outcome of neonatal mortality on days 3 through 27. The delivery and postdelivery data sets were censored for deaths occurring prior to grouping time points and missing data so that only surviving neonates with complete data were included.

To build and validate predictive models, split sampling was used to set aside sections of the data for uncertainty estimation and model validation of predictive accuracy. The models considered were logistic regression and 5 machine learning models (SVM [support vector machine with radial basis function kernel], EN [logistic elastic net], NN [neural network], GBE [gradient boosted ensemble], and RF [random forest]). Data management was completed using SAS 9.4 software (SAS Institute Inc), and the model building was completed using the scikit-learn Python module. Graphics were completed using R 4.0.2 (R Project for Statistical Computing). All models except logistic regression were tuned using 10-fold cross-validation on training data, and then each tuned model was applied to the test data for a predictive accuracy assessment. For assessment of the consistency of accuracy, the tuning was repeated on training plus test data, and tuned models were applied to the validation data. The predictive accuracy was assessed using the area under the curve (AUC) of the receiver operating characteristic (ROC) curves. Because the result from any accuracy assessment using randomly split data is random, the entire analysis was repeated within each of 10 mutually exclusive data subsets for each scenario. This enabled us to have 10 assessments of accuracy for each model within each scenario, allowing an assessment of the uncertainty in the estimated accuracy for all models. Paired *t* tests were used on these 10 estimates of accuracy to compare models in order to descriptively assess whether or not the models within a scenario were discernably different in light of the uncertainty. The process of building and validating the best predictive machine learning model and using the results to build a modified logistic regression model for mortality risk scoring is described in the eAppendix in the Supplement.

## Results

**Figure 1.**
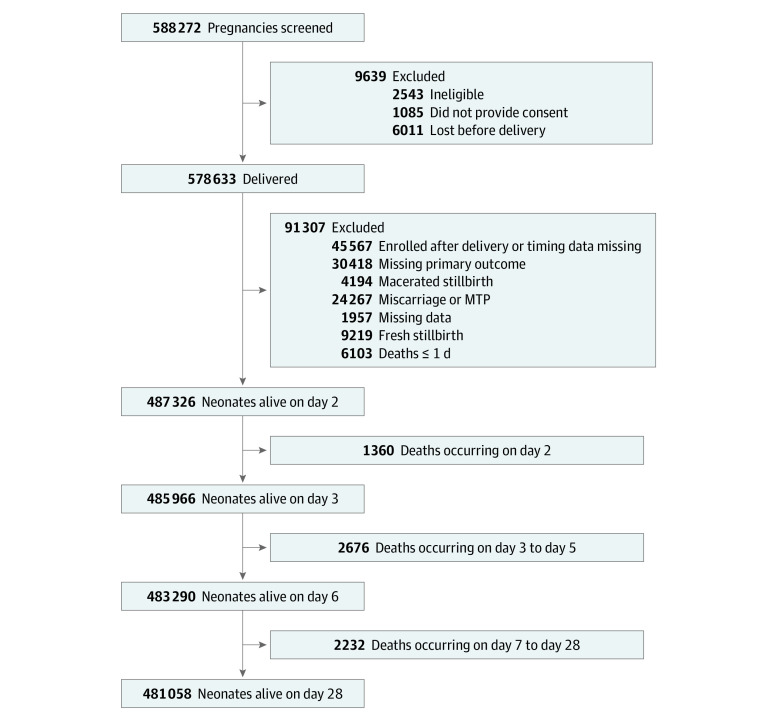
Participant Flow Diagram The flow diagram denotes the number of participants for each data set prior to the removal of missing covariate data. The data sets were censored for both deaths occurring before the time points and missing data. Ineligible participants included those who were enrolled early and later found not to be pregnant and those who were residing outside the study clusters. 502 648/578 633 deliveries had outcome data, 487 642/502 648 deliveries had complete predictor data for prenatal scenario, 487 537/502 648 deliveries had complete predictor data for predelivery scenario, 469 516/487 326 neonates alive on day 2 had complete predictor data for delivery/day 1 scenario, and 468 356/485 966 neonates alive on day 3 had complete predictor data for postdelivery/day 2 scenario.

After the removal of missing data and deaths before grouping time points, the prenatal data set contained 487 642 neonates, the predelivery data set contained 487 537 neonates, the delivery/day 1 data set contained 469 516 neonates, and the postdelivery/day 2 data set contained 468 356 neonates (Figure 1). The sex distribution of the neonates was 51.6% male and 48.4% female in the prenatal data set. Baseline maternal and neonatal variables vary slightly for each of the 4 data sets owing to censoring (Table 1). The sample sizes for each subset before splitting into training, test, and validation data sets also vary slightly, which reflects variation owing to missing data (eTable 1 in the Supplement).

**Table 1.** Baseline Maternal and Neonatal Variables

Variable	Prenatal (n = 487 642)		Predelivery (n = 487 537)	Delivery/day 1 (n = 469 516)		Postdelivery/day 2 (n = 468 356)	Scenario
**Continuous variables, mean (SD)**							
Gestational age at enrollment, wk	20.2 (9.2)	20.2 (9.2)			20.2 (9.3)	20.2 (9.3)	Prenatal
Parity, No.	1.8 (2.1)	1.8 (2.1)			1.7 (2.1)	1.7 (2.1)	Prenatal
Cluster mortality, rate^a^	0.04 (0.02)	0.04 (0.02)			0.04 (0.02)	0.04 (0.02)	Prenatal
Maternal age, y	24.8 (5.3)	24.8 (5.3)			24.8 (5.3)	24.8 (5.3)	Prenatal
Gestational age, wk	38.6 (3.5)^b^	38.6 (3.5)^b^			38.7 (3.3)	38.7 (3.3)	Delivery/day 1
Birth weight, g	2899 (505)^c^	2899 (505)^c^			2914 (480)	2916 (478)	Delivery/day 1
**Categorical variables, No. (%)**							
Site							Prenatal
DRC	31 141 (6.4)	31 138 (6.4)			29 595 (6.3)	29 553 (6.3)	
Zambia	60 159 (12.3)	60 157 (12.3)			58 556 (12.5)	58 489 (12.5)	
Guatemala	73 901 (15.2)	73 901 (15.2)			72 295 (15.4)	72 119 (15.4)	
Belagavi, India	101 385 (20.8)	101 350 (20.8)			97 913 (20.9)	97 671 (20.9)	
Pakistan	76 273 (15.6)	76 218 (15.6)			71 102 (15.1)	70 793 (15.1)	
Nagpur, India	77 774 (15.9)	77 769 (16.0)			75 612 (16.1)	75 354 (16.1)	
Kenya	67 009 (13.7)	67 004 (13.7)			64 443 (13.7)	64 377 (13.7)	
Maternal variables							
Age (categorical), y							Prenatal
<20	63 976 (13.1)	63 963 (13.1)			61 695 (13.1)	61 562 (13.1)	
20-35	401 172 (82.3)	401 084 (82.3)			386 461 (82.3)	385 491 (82.3)	
>35	22 494 (4.6)	22 490 (4.6)			21 360 (4.5)	21 303 (4.5)	
Education (categorical)							Prenatal
None	111 608 (22.9)	111 558 (22.9)			105 057 (22.4)	104 716 (22.4)	
Primary	143 875 (29.5)	143 853 (29.5)			138 867 (29.6)	138 549 (29.6)	
Secondary	197 542 (40.5)	197 510 (40.5)			191 767 (40.8)	191 354 (40.9)	
University	34 617 (7.1)	34 616 (7.1)			33 825 (7.2)	33 737 (7.2)	
Birth order							Prenatal
First	482 984 (99.0)	482 885 (99.0)			465 612 (99.2)	464516 (99.2)	
Second	4615 (0.9)	4609 (0.9)			3875 (0.8)	3811 (0.8)	
Third	42 (0.0)	42 (0.0)			29 (0.0)	29 (0.0)	
Fourth	1 (0.0)	1 (0.0)			0 (0.0)	0 (0.0)	
≥1 prenatal visit	474 530 (97.4)	474 433 (97.4)			457 467 (97.5)	456 338 (97.5)	Predelivery
Antepartum hemorrhage	5625 (1.2)	5621 (1.2)			3856 (0.8)	3797 (0.8)	Predelivery
Hypertension	12 798 (2.6)	12 793 (2.6)			11 501 (2.5)	11 407 (2.4)	Predelivery
Suspected sepsis	2142 (0.5)	2141 (0.5)			1902 (0.4)	1892 (0.4)	Predelivery
Eclampsia	222 (0.0)	222 (0.0)			192 (0.0)	191 (0.0)	Predelivery
Antenatal corticosteroids	9228 (2.6)	9227 (2.6)			8544 (2.5)	8463 (2.5)	Predelivery
Hospitalization	3790 (0.8)	3790 (0.8)			3502 (0.8)	3492 (0.8)	Postdelivery/day 2
Antibiotics	195 383 (48.6)	195 368 (48.6)			187 268 (48.3)	186 604 (48.3)	Postdelivery/day 2
**Delivery variables**							
Attendant							Predelivery
Physician	177 644 (36.4)	177 605 (36.4)			170 942 (36.4)	170 362 (36.4)	
Nurse/midwife	180 191 (37.0)	180 181 (37.0)			174 874 (37.2)	174 562 (37.3)	
TBA	105 025 (21.5)	105 016 (21.5)			100 981 (21.5)	100 775 (21.5)	
Family/self/other	24 735 (5.1)	24 735 (5.1)			22 719 (4.8)	22 657 (4.8)	
Location							Predelivery
Hospital	219 777 (45.1)	219 767 (45.1)			211 746 (45.1)	211 101 (45.1)	
Clinic/health center	146 058 (30.0)	146 034 (30.0)			141 705 (30.2)	141 417 (30.2)	
Home	121 743 (25.0)	121 736 (25.0)			116 065 (24.7)	115 838 (24.7)	
Lie							Predelivery
Transverse	306 (0.5)	306 (0.5)			289 (0.5)	286 (0.5)	
Oblique	129 (0.2)	129 (0.2)			115 (0.2)	115 (0.2)	
Breech	823 (1.3)	823 (1.3)			717 (1.1)	715 (1.1)	
Vertex	64 073 (98.1)	64 071 (98.1)			62 169 (98.2)	62 026 (98.2)	
Multiple birth	9251 (1.9)	9243 (1.9)			7957 (1.7)	7846 (1.7)	Delivery/day 1
Mode							Delivery/day 1
Vaginal	413 453 (84.8)	413 364 (84.8)			396 606 (84.7)	396 704 (84.7)	
Vaginal, assisted	4753 (1.0)	4750 (1.0)			4311 (0.9)	4284 (0.9)	
CD	69 425 (14.2)	69 418 (14.2)			67 599 (14.4)	67 368 (14.4)	
Obstructed labor	40 540 (8.3)	40 527 (8.3)			36 938 (7.9)	36 760 (7.9)	Delivery/day 1
**Neonatal variables**							
Sex							Delivery/day 1
Male	251 197 (51.6)	251 148 (51.6)			241 240 (51.4)	240 561 (51.4)	
Female	236 080 (48.4)	236 026 (48.4)			228 240 (48.6)	227 759 (48.6)	
Bag and mask resuscitation	19 669 (4.1)	19 649 (4.1)			16 692 (3.6)	16 254 (3.5)	Delivery/day 1
Congenital anomalies	1084 (0.5)	1084 (0.5)			677 (0.3)	658 (0.3)	Delivery/day 1
Hospitalization	9650 (2.2)	9648 (2.2)			9474 (2.1)	9315 (2.1)	Postdelivery/day 2
Antibiotics	20 740 (5.8)	20 736 (5.7)			19 178 (5.5)	18 726 (5.4)	Postdelivery/day 2
Cord care	83 172 (77.2)	83 172 (77.2)			81 661 (78.8)	81 416 (78.8)	Postdelivery/day 2
Medicinal cord care	52 076 (20.6)	52 076 (20.6)			51 110 (20.9)	50 950 (20.9)	Postdelivery/day 2

Abbreviations: CD, cesarean delivery; DRC, Democratic Republic of Congo; TBA, traditional birth attendant.

^a^ Cluster perinatal mortality is the rate of perinatal mortality within each distinct geographical area (cluster) of the sites as defined by the Global Network.

^b^ Missing 0.40% of data.

^c^ Missing 1.13% of data.

**Figure 2.**
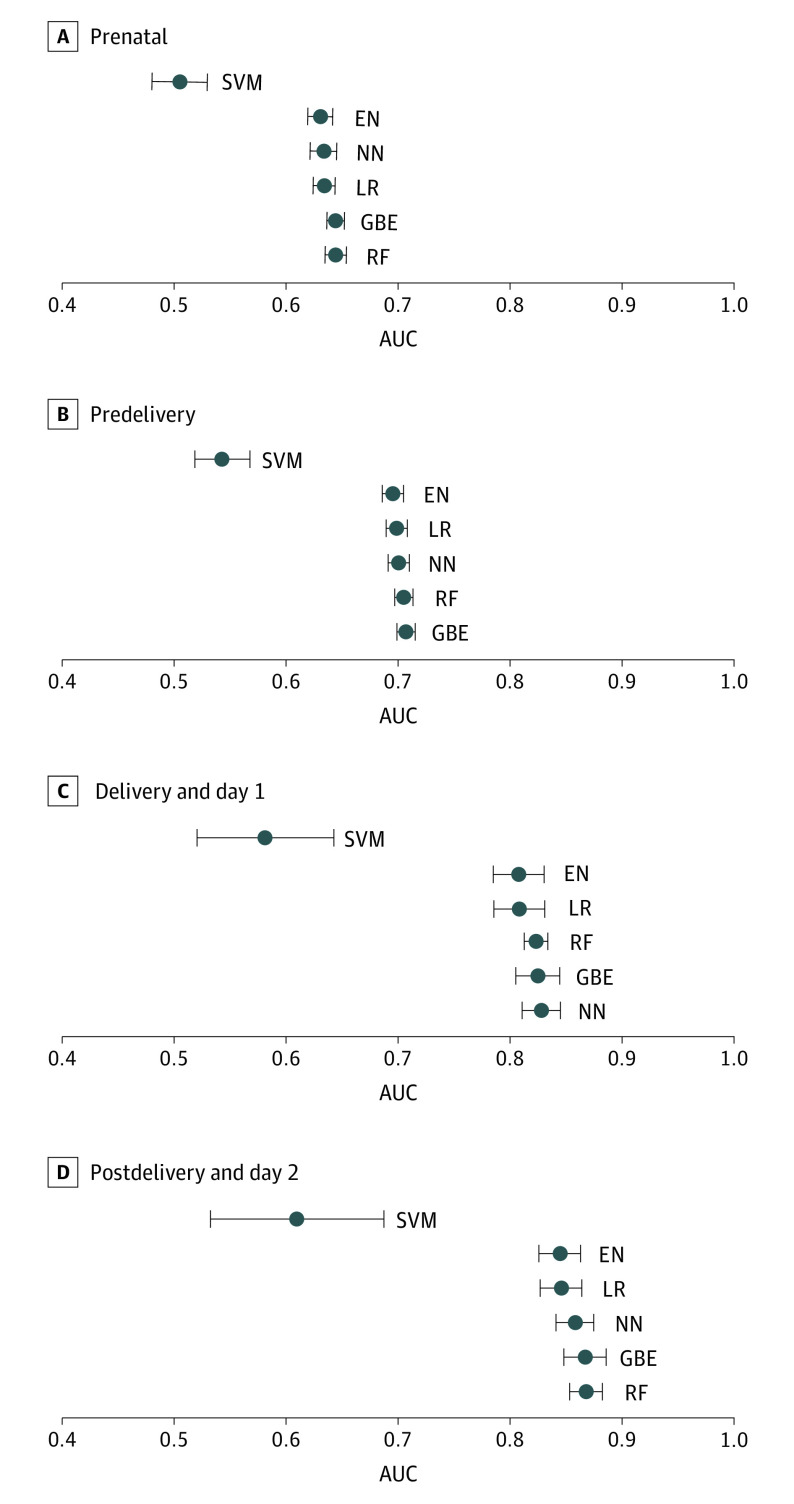
Mean (95% CI) for Validation AUC by Scenario for Outcomes of Intrapartum Stillbirth and Neonatal Mortality EN indicates logistic elastic net; GBE, gradient boosted ensemble; NN, neural network; RF, random forest; and SVM, support vector machine with radial basis function kernel.

Models using either only prenatal or prenatal and predelivery variables had predictive accuracy for intrapartum stillbirth and neonatal mortality of AUC values 0.71 or less (Figure 2). The analysis of models for intrapartum stillbirth showed that all prenatal models had AUC values of 0.63 or less and all predelivery models had AUC values of 0.72 or less (eFigure 1 in the Supplement). Cluster perinatal mortality was the most important predictor of intrapartum stillbirth in the prenatal data set (AUC, 0.60) and antepartum hemorrhage was the most important predictor in the predelivery data set (AUC, (0.56) (eTable 2 in the Supplement). Other important predictors of intrapartum stillbirth in the prenatal data set were gestational age at enrollment, maternal age, birth order, and parity. Other important predictors of intrapartum stillbirth in the predelivery data set were cluster perinatal mortality; gestational age at enrollment; hypertension, severe pre-eclampsia, or eclampsia; and maternal age.

**Figure 3.**
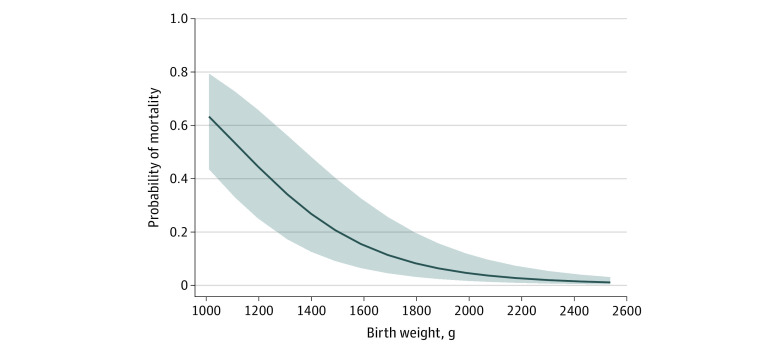
Probability of Mortality as a Function of Birth Weight, Delivery/Day 1 Scenario

The predictive models based on the data sets that included delivery/day 1 and postdelivery/day 2 variables had good predictive accuracy for neonatal mortality, with 5 of the 6 models having AUCs above 0.80. Birth weight was the most important predictor in both the delivery/day 1 and postdelivery/day 2 scenarios, with independent predictive ability of AUC 0.78 and 0.76, respectively. The increase in probability of mortality with decreasing in birth weight occurred in both the delivery/day 1 and postdelivery/day 2 scenarios (Figure 3; eFigure 2 in the Supplement). Bag and mask resuscitation, gestational age, cluster perinatal mortality rate, and maternal age were the other top predictors for the delivery/day 1 scenario. Conditions requiring hospitalization, antibiotics, gestational age, and bag and mask resuscitation were the other top predictors for the postdelivery/day 2 scenario. The addition of these other top predictors resulted in increases in the AUCs (0.83 and 0.87 for the delivery/day 1 and postdelivery/day 2 scenarios, respectively) relative to birth weight alone.

For models assessing the outcomes of stillbirth and neonatal mortality, the pairwise paired *t* test showed that there were statistically insignificant differences between AUC values illustrated by considerable overlap of confidence intervals (Figure 2). However, gradient boosted ensemble and random forest models were consistently among the best-performing models. Even though the logistic regression model was not the best-performing model in any scenario, the AUC of the logistic regression model was not significantly different than the top-performing models.

The sequential addition of variables was done to identify the individual relative contribution of each variable to AUC for combined outcomes of intrapartum stillbirth and neonatal mortality based on validation data set (Table 2) and to develop a risk scoring system for stillbirth and neonatal mortality. Table 2 lists the changes in AUC as predictors are added to the model using the ordering of predictor importance (see eAppendix in the Supplement for more details about predictor importance). A modified logistic regression model based on findings from the best machine learning model as well as results from a variable selection study using logistic regression and the least absolute shrinkage and selection operator (LASSO) method for identification of potential interactions was fit to create a risk scoring system for the delivery/day 1 and postdelivery/day 2 data (eTable 3 in the Supplement). Using risk scores calculated from this risk scoring system, a logistic regression model was fit to the total risk scores in order to derive a formula for predicting the probability of mortality given the calculated risk score. The logistic model for neonatal mortality risk scoring had an AUC value on validation data equal to 0.809 (97% of the best model) for the delivery/day 1 scenario and 0.845 (97% of the best model) for the postdelivery/day 2 scenario (eTable 4 in the Supplement).

**Table 2.** Top Predictors by Scenario (Intrapartum Stillbirth and Neonatal Mortality Outcome)^a^

Rank	Predictor	AUC	AUC increase
**Delivery/day 1**			
1	Birth weight	0.776	NA
2	Bag and mask resuscitation	0.814	+0.039
3	Gestational age	0.817	+0.003
4	Cluster perinatal mortality	0.816	−0.001
5	Maternal age	0.820	+0.004
6	Parity	0.822	+0.002
7	Gestational age at enrollment	0.822	0
8	Antepartum hemorrhage	0.823	+0.001
9	Multiple birth	0.824	+0.001
10	Hypertension/severe pre-eclampsia/eclampsia^b^	0.822	−0.002
11	Antenatal corticosteroids	0.823	+0.001
12	Birth order	0.822	−0.001
13	Suspected maternal sepsis^c^	0.823	+0.001
14	Neonatal sex	0.827	+0.004
15	Obstructed labor	0.829	+0.002
**Postdelivery/day 2**			
1	Birth weight	0.763	NA
2	Neonatal hospitalization	0.813	+0.050
3	Neonatal antibiotics	0.845	+0.032
4	Gestational age	0.842	−0.003
5	Bag and mask resuscitation	0.852	+0.010
6	Cluster perinatal mortality	0.865	+0.013
7	Gestational age at enrollment	0.867	+0.002
8	Maternal age	0.870	+0.003
9	Parity	0.870	0
10	Multiple birth	0.870	0
11	Antenatal corticosteroids	0.871	+0.001
12	Maternal education: none	0.871	0
13	Hospital delivery	0.872	+0.001
14	Maternal antibiotics	0.871	−0.0001
15	Delivery by physician	0.872	+0.001

Abbreviations: AUC, area under the curve; NA, not applicable.

^a^ Predictors are added consecutively using gradient boosted ensemble model; then AUC calculated. The order used in this table was the order of importance assigned by the predictive model assessment of important predictors.

^b^ Hypertensive disease/severe pre-eclampsia/eclampsia defined as blood pressure >140/90 mm Hg, proteinuria, and seizures.

^c^ Suspected maternal sepsis defined as fever with pelvic pain and abnormal vaginal discharge (foul smelling or presence of pus).

## Discussion

This cohort study found that predictive models using only prenatal or prenatal and predelivery variables had predictive accuracy for intrapartum stillbirth and neonatal mortality of AUC values of 0.71 or less. We identified that a better neonatal mortality risk prediction could be made when variables obtained immediate postdelivery and up to 2 days after birth were included in the models, with AUCs increasing up to 0.87. Birth weight was identified as the most important predictor for neonatal mortality among all variables considered. The contribution of other predictors to the AUC increase was relatively minor.

Many studies have analyzed variables associated with increased risk for stillbirths or neonatal mortality, but only 1 study^16^ used a relatively large group of neonates to develop predictive models. As found in the current study, low predictive accuracy (AUC = 0.58, 95% CI = 0.56–0.59) using prenatal variables was reported in a study based on 10 sites from 3 countries in South Asia (N = 49 632).^16^ Similar to the findings of the current study, the predictive accuracy of the model improved when postdelivery variables were included (AUC = 0.83, 95% CI = 0.79–0.86), but only logistic regression modeling was applied to develop the model, and the results were not validated with an independent sample. The current results are consistent with the data from a large prospective neonatal database of participants living in a high-income country, which showed that inclusion of delivery variables (especially birth weight) results in better accuracy for predicting neonatal mortality than models with only prenatal variables.^15^

Some of the important predictors in the current study have been reported to be associated with an increased risk for neonatal mortality in simple association analyses. In a pooled analysis of data from low- and middle-income countries, small for gestational age and prematurity were found to be associated with increased risk of neonatal mortality in association (bivariate) analysis, although birth weight was not specifically analyzed.^17^ Inferences from pooled analyses can raise questions of quality, accuracy, and generalizability.^18^ Additionally, vital registries from low- and middle-income countries are of questionable accuracy.^19^ The novel finding of the current study that birth weight is the most important variable for predicting the risk of neonatal mortality provides the strongest evidence based on a high-quality large prospective population-based database from resource-limited settings. To our knowledge, the current study is the largest study that uses a quantitative approach for risk prediction of stillbirth and neonatal mortality of data from low- and middle-income countries. This study identifies the contribution of birth weight as a continuous variable to arrive at its independent predictive ability for risk of neonatal mortality. In studies of association with increased risk of all-cause stillbirth or neonatal mortality, many variables have been identified, but these analyses were limited to descriptive analyses. Intrapartum stillbirth and early neonatal mortality have overlapping causes (in contrast to early stillbirth^20^), so we have included only intrapartum stillbirth in the present study. Adjusted analysis based on an earlier cohort of the GN-MNHR database indicated associations between maternal age younger than 20 or older than 35 years, lower maternal education, 0 parity or 3 parity greater than 3, and no prenatal care with all-cause stillbirth.^21,22^ Using other databases, other factors associated with all-cause stillbirth were poverty, parity of 5 or more, prematurity, low birth weight, and previous stillbirth.^20^ Factors associated with neonatal mortality also included maternal age, education, parity, multiple gestations, birth order, suspected maternal sepsis, antepartum hemorrhage, eclampsia, and obstructed labor.^23,24,25^ Also, in a large pooled analysis of cross-sectional data from 57 low- and middle-income countries (N = 464 728), antenatal care was associated with decrease in neonatal mortality in univariate analyses.^26^

In large modeling studies from high-income countries of extremely preterm^15,27^ or less than 2000 g neonates^28^ admitted to neonatal intensive care units, birth weight was among the most important common factors associated with hospital mortality. With the model derived from a high-income country, birth weight was also found to be a factors associated with hospital mortality when it was applied to a sample of 550 neonates from a single center in The Gambia.^28^ In another study from a developed country database of extreme preterm neonates, birth weight and gestational age were found to be equally associated with 2-year mortality.^29^ Gestational age was also among the top factors associated with mortality in the earlier developed country database studies.^15,27^ However, in the current study, birth weight was found to be a better predictor of neonatal mortality than gestational age. This could be related to estimation errors of gestational age based on the last menstrual period and limited availability of first-trimester ultrasonography-based dating confirmation.^30^ Small for gestational age (not specifically birth weight) has been associated with higher risk of perinatal and neonatal mortality and morbidity in data from high-income countries in bivariate analysis.^31^ Similar to the current study, neonatal sex, resuscitation, and antenatal corticosteroids have been factors associated with neonatal mortality in data from high-income countries.^32,33,34^

### Limitations

This study used an established database with data that are population-based and prospectively collected with multiple quality assurance checks. The study was hypothesis-driven with only prespecified analyses performed. This large sample size study was adequately powered for prediction of the risk for intrapartum stillbirth and neonatal mortality in the represented resource-limited settings in low- and middle-income countries. Because of the large sample size, we were able to evaluate rigorously the machine learning predictive models. Predictive accuracy was assessed on test and validation data sets to check for consistency in predictive accuracy estimates, and the modeling building process was completed 10 times to quantify uncertainty in predicted model accuracy. Although generalizability of the results could be questioned, the results are likely to be pertinent to many communities in resource-limited regions similar to those of the study settings. There is a possibility of potential confounders like health status, availability of care, interventions, and health policy that were not captured. Nonetheless, as the study results are based on a large number of participants over 9 years, the potential impact of confounders on the study result should be low. Additionally, the use of the database did not include high-definition data, including stratification of individual risk factors as per illness severity, extensive laboratory test results, or details of treatments received and clinical response. As the risk score is intended to be useful for all health care professionals, adding more complexity to the scoring method by including variables that need a higher level of training and resources might have reduced the ease of application and usability of the score. However, incorporating additional variables could have improved the predictive accuracy, especially for the prenatal models. The gestational age variable would be prone to estimation errors depending on the mother’s accounting of the last menstrual period and the availability of more accurate assessments such as first-trimester ultrasonography. Intrapartum and antepartum stillbirth differentiation can be associated with identification errors, but training of the health care professionals and several quality checks were made to minimize this error.

## Conclusions

In the current study, prediction of the risk of intrapartum stillbirth alone or in combination with neonatal mortality based on prenatal or predelivery data had predictive accuracy of AUC values of only 0.72 or less. The best risk prediction for neonatal death was only achieved after including delivery and early neonatal variables, which can be used to identify neonates at the highest risk for mortality who may need specialized care. Birth weight was by far the most important predictor for neonatal mortality, while the contribution of other variables was relatively minor. Mortality risk–based triage and referral could be tested as a strategy to reduce the burden of neonatal deaths in resource-limited settings. Given these findings, prenatal and predelivery data are not sufficient to develop strategies to identify those who are at a high risk of perinatal mortality and require advanced care at birth and referral. Birth weight could be prioritized in the identification of neonates at risk for dying. Predelivery estimation of birth weight could be evaluated as a strategy for predelivery triage and referral.

## Notes

Supplement. **eMethods.** **eTable 1.** Subset Sample Sizes **eTable 2.** Top Predictors by Scenario (Fresh Stillbirth Outcome) **eTable 3.** Risk Score Model Coefficients and Calculation (Neonatal Mortality Outcome) **eTable 4.** Mortality Probability Functions for the Total Risk Score (Neonatal Mortality Outcome) **eFigure 1.** Mean (95%CI) for Validation AUC by Scenario for Outcomes of Fresh Stillbirth **eFigure 2.** Probability of Mortality as a Function of Birthweight, Post-delivery/Day-2 Scenario **eReferences.**
