# Supplementary material for: Predictive Modeling for Perinatal Mortality in Resource-Limited Settings
Source: JAMA Netw Open. 2020 Nov 18;3(11):e2026750. doi: 10.1001/jamanetworkopen.2020.26750 (PMC7675108; doi:10.1001/jamanetworkopen.2020.26750)
Supplement: Supplement. — eMethods. eTable 1. Subset Sample Sizes eTable 2. Top Predictors by Scenario (Fresh Stillbirth Outcome) eTable 3. Risk Score Model Coefficients and Calculation (Neonatal Mortality Outcome) eTable 4. Mortality Probability Functions for the Total Risk Score (Neonatal Mortality Outcome) eFigure 1. Mean (95%CI) for Validation AUC by Scenario for Outcomes of Fresh Stillbirth eFigure 2. Probability of Mortality as a Function of Birthweight, Post-delivery/Day-2 Scenario eReferences. [file jamanetwopen-e2026750-s001.pdf]

Supplemental Online Content

Shukla VV, Eggleston B, Ambalavanan N, et al. Predictive modeling for perinatal Mortality in resource-limited settings. *JAMA Netw Open*. 2020;3(11):e2026750. doi:10.1001/jamanetworkopen.2020.26750

eMethods.
eTable 1. Subset Sample Sizes
eTable 2. Top Predictors by Scenario (Fresh Stillbirth Outcome)
eTable 3. Risk Score Model Coefficients and Calculation (Neonatal Mortality Outcome)
eTable 4. Mortality Probability Functions for the Total Risk Score (Neonatal Mortality Outcome)
eFigure 1. Mean (95%CI) for Validation AUC by Scenario for Outcomes of Fresh Stillbirth
eFigure 2. Probability of Mortality as a Function of Birthweight, Post-delivery/Day-2 Scenario
eReferences.

This supplemental material has been provided by the authors to give readers additional information about their work.

© 2020 Shukla VV et al. *JAMA Network Open.*

eMethods

Data preparation

The GN-MNHR database utilizes data edits, including inter- and intra-form consistency checks, upon data entry locally and by the data coordinating center (RTI International, Research Triangle Park, NC, USA) as measure to ensure quality data. In addition to these database operations, additional data preparation was completed for analysis using machine learning models. An analysis involving machine learning models requires continuous predictors to be on the same variability scale and requires multi-level categorical predictors to be transformed into a numeric indicator variable (binary categorical predictor) or a set of numeric indicator variables (multi-level categorical predictors). For this analysis, continuous variables were standardized to a mean equal to 0 and a standard deviation of 1. For all models, once a categorical variable was transformed into a set of numerical indicator variables, one of the numerical indicator variables was removed to create a reference cell coding scheme.

Statistical dataset construction

The risk factors were added sequentially into four scenario data sets: 1) prenatal (variables till first prenatal care visit), 2) pre-delivery (variables till just before delivery), 3) delivery and day-1 (delivery/day-1), and 4) post-delivery through day-2 (post-delivery/day-2). Day-0 was defined as the calendar day of birth. Day-1 and day-2 were defined as the subsequent calendar days. We evaluated mortality outcomes using potential risk factor sets with sequentially additional variables to determine whether additional potential risk factors improved outcome predictive accuracy.[1] The first two scenario models (prenatal and pre-delivery) evaluated the outcome of fresh stillbirth and neonatal mortality. The third scenario model (delivery/day-1) evaluated the

© 2020 Shukla VV et al. *JAMA Network Open.*

outcome of neonatal mortality on days 2 through day-27. The fourth scenario model (post-delivery/day-2) evaluated the outcome of neonatal mortality on days 3 through day-27. Within each scenario records of neonates with missing data among the predictors were excluded from the analyses so that only neonates with complete data were included in the analyses. Also, the delivery/day-1 and post-delivery data sets were censored for deaths occurring prior to grouping time points so that only surviving neonates were included. The full data had 502,648 records with outcomes. The prenatal analysis removed 15,006 records due to missing data among the predictors, while the pre-delivery analysis removed 15,111 records due to missing data among the predictors. For the delivery/day-1 analysis, 487,326 records were available after censoring for prior death, of these 17,814 records were removed due to missing data among the predictors. Finally, for the post-delivery/day-2 analysis, 485,966 records were available after censoring for prior death, of these 17,610 records were removed due to missing data among the predictors.

Predictive model building process

The process of building and validating the predictive models followed the approach of Eggleston et al. with the addition of more model assessment procedures due to more data being available.[2] As noted above, the analysis utilized 4 scenario datasets, each representing a set of predictors available at a specific time relative to delivery. Each scenario dataset was analyzed using the same process described here.

For a given scenario, we first randomly divided each scenario data sets into ten subgroups, and each subgroup was divided again randomly into three analysis data sets, training (60%), test (20%), and validation (20%) data sets.[2] After the data splitting, a set of conventional and

© 2020 Shukla VV et al. *JAMA Network Open.*

advanced machine learning predictive models were fit (and tuned if possible) using the training analysis dataset within all 10 data subgroups for a given scenario. The models fit were logistic regression and advanced machine learning models (SVM (support vector machine with radial basis function kernel), EN (logistic elastic net), NN (neural network), GBE (gradient boosted ensemble), and RF (random forest)). Data management was completed using SAS 9.4 software (SAS Institute, Cary, NC),[3] and the model building was completed using the scikit-learn Python module.[4] Graphics were completed using R 4.0.2.[5] The scikit-learn LogisticRegression() function was used for logistic regression. The scikit-learn SGDClassifier() function with log loss and elasticnet penalty was used for the elastic net. The scikit-learn MLPClassifier() function was used to fit the neural network. The sci-kit learn RandomForestClassifier() function was used to fit the Random Forest. The sci-kit learn GradientBoostingClassifier() function was used to fit the Grandient Boosting ensemble. Finally, the sci-kit learn SVC() function was used to fit the support vector machine. The sci-kit learn GridSearchCV() function was used to tune the models, The sci-kit learn roc_auc_score() function as used to estimate AUC of ROC. The feature_importances attribute of fitted models was used to assess relative importance of predictors. All models except logistic regression were tuned using 10-fold cross-validation on training data, and then each tuned model was applied to the test data for a predictive accuracy assessment.[2] For assessment of the consistency of accuracy, the tuning was repeated on training plus test data, and tuned models were applied to the validation data. The predictive accuracy was assessed using the area under the curve (AUC) of the receiver operating characteristic (ROC) curves.[6] The models considered in this manuscript were selected because the authors were agnostic concerning what model would be best suited for the prediction of neonatal mortality and each model uniquely uses the information in the data to predict neonatal mortality.

© 2020 Shukla VV et al. *JAMA Network Open.*

Due to the splitting of the data into 10 subsets of training/test/validation data, a second round of validation was also performed within each of the 10 subsets by retuning the models on the training plus test data and assessing predictive accuracy using the validation data. As with the first validation, the predictive accuracy was assessed using the area under the curve (AUC) of the receiver operating characteristic (ROC) curves. This process of tuning on training data using 10-fold cross-validation, followed by the assessment on test data, and re-evaluated a second time on validation data after tuning on training plus test data, repeated ten times on separate subsets, allowed the authors to get a good picture about which model was consistently better than other models considered and assess the stability of the predictive accuracy.

Because the entire analysis was repeated with each of the ten subsets for each scenario, the authors could calculate for each tuned model an average AUC on test data and a separate average AUC on validation data, the standard error of AUC on test and validation data, as well as compute paired t-tests for all possible combinations of model comparisons. For these comparisons, the null hypothesis was no differences in the predictive accuracy across each pair of models represented in the paired t-tests. No adjustments for multiple comparisons were made. Paired t-tests were only completed on the AUCs calculated using validation data. Completing the paired t-tests, allowed the authors to recognize the variability present in the validation-based AUC values and use this information to interpret better the ordering of average validation based AUCs.

Potential improvement of logistic regression model

© 2020 Shukla VV et al. *JAMA Network Open.*

Once validation-based AUC values and paired t-tests results were reviewed a best model was identified for use in developing a potentially improved logistic regression model within a given scenario, if the validation-based AUC was at least 0.80. Given that the best models in the two scenarios that had sufficient validation-based AUC values were tree-based ensemble models, this additional analysis involved identifying the top 15 important predictors within the model using the GINI importance measure.[7] These top 15 predictors were included in a set of potentially important predictors for inclusion in the potentially improved logistic regression model. In addition to using the top 15 predictors from the best model, the least absolute shrinkage and selection operator (LASSO) method was used to investigate the value of any two-way interactions between predictors in the original logistic regression model excluding site variables. If an interaction term was identified using the least absolute shrinkage and selection operator (LASSO) as a potentially valuable interaction term, the interaction term was added to list of potentially important predictors. Additional predictors were added to the list of potentially important predictors so that no interaction term was present without main effect terms being added as well. With this full set of potentially important predictors, modified logistic regression models were built as surrogate models for interpretation and if useful as a basis for developing a portable risk scoring system that could be fully described in print. The scoring system represented by eTable3 and eTable4 are intended for use as an online calculator that is easy to use by healthcare providers. In both the delivery/day-1 scenario and the post-delivery/day-2 scenario, the resulting modified logistic regression model had comparable predictive accuracy relative to the best model and utilized less variables resulting in an easier application of the model. Site variables were excluded from this component of modeling fitting to increase generalizability.

© 2020 Shukla VV et al. *JAMA Network Open.*

Development of portable risk scoring system

For the delivery/day-1 and post-delivery/day-2 scenarios, the modified logistic regression model was used to translate measures of risk into probabilities of mortality by creating risk score weights from the parameter coefficients, eTable3. These risk score weights were created by multiplying the parameter coefficients by 10 and rounding off to the nearest tenth decimal place. These risk score weights were used to create a total risk score by taking the product of each risk score weight and risk measure value and then summing up all the products. The total risk scores were used to generate a logistic regression model that would translate the total risk score into a probability of mortality, eTable4. The logistic regression models, reported in eTable4, are somewhat redundant for simply calculating the probability of mortality, but the calculated total risk score is on a better scale than the linear combination of covariate value by parameter estimate and the logistic regression models in eTable4, which use the total risk score as the only covariate, shows how changes in the total risk score change the probability of mortality. The predictive accuracy of the models was assessed using the entire validation data to evaluate the quality of the logistic regression-based prediction model relative to the machine learning models.

Sample size justification

One rule of thumb for predictive modeling is 10 events per variable; however, machine learning models require a relatively large sample size for good predictive accuracy. For some machine learning models, Ploeg et al. have shown via simulation that an events per variable ratio of >200 may be needed for achieving stable estimates of predictive accuracy.[8] Depending on the scenario, the data used in this analysis was clearly deemed sufficiently large either because the

© 2020 Shukla VV et al. *JAMA Network Open.*

events per variable ratio was greater than 200, or the deemed sufficiently large because the events per variable ratio was greater than 10 given we split the data in such a way that models were validated twice and estimates of uncertainty in predictive accuracy were quantified. Before data splitting, the event rate per variable ratio for the prenatal analysis was 1477 for the combined outcome of total fresh stillbirths and total neonatal deaths. After setting aside 60% of the data for training, the training data set had an event per variable ratio of 886. Before data splitting, the event rate per variable ratio for the pre-delivery analysis was 738 for the combined outcome of total fresh stillbirths and total neonatal deaths. After setting aside 60% of the data for training, the training data set had an event per variable ratio of 443. Therefore, the available sample size was considered large enough to adequately train models on the prenatal and pre-delivery data.

Before data splitting, the event rate per variable ratio for the delivery/day-1 and the post-delivery/day-2 analyses was 152 and 104 respectively for the outcome of neonatal deaths. The training data sets of 60% of total data had event per variable ratios of 91 and 62 respectively. Although the event per variable ratios for the delivery (day-1) and post-delivery (day-2) analyses were not greater than 200, they were greater than the rule of thumb of 10 events per variable. Our method of repeating the model fitting process 10 times to estimate predictive accuracy on test data, and then repeating the process again to estimate predictive accuracy on validation data was considered sufficient to quantify the variability in overall predictive accuracy assessments and identify cases where the model building process was unreliable.

© 2020 Shukla VV et al. *JAMA Network Open.*

eTable1: Subset Sample Sizes

Data Subset	Prenatal	Pre-delivery	Delivery/day-1	Post-delivery/day-2
1	48,730	48,714	46,835	46,732
2	48,779	48,768	46,891	46,773
3	48,772	48,761	46,956	46,829
4	48,757	48,746	46,923	46,808
5	48,818	48,811	47,024	46,916
6	48,787	48,774	47,070	46,949
7	48,811	48,803	47,023	46,908
8	48,749	48,739	47,009	46,881
9	48,718	48,707	46,842	46,721
10	48,721	48,714	46,943	46,839

*Variation in subset size reflects variation in the number of records removed due to missing data. Records were removed for missing data after subset assignment, due to missing values investigation was done in Python, while subset assignment was completed in SAS.

© 2020 Shukla VV et al. *JAMA Network Open.*

eTable2: Top Predictors by Scenario (Fresh Stillbirth Outcome) *

Rank	Predictor	AUC	AUC increase
Prenatal			
1	Cluster perinatal mortality	0.602	NA
2	Gestational age at enrollment	0.610	+0.008
3	Maternal age	0.613	+0.003
4	Birth order	0.621	+0.008
5	Parity	0.630	+0.009
6	Maternal education – none	0.634	+0.004
7	Maternal education – primary	0.635	+0.001
8	Maternal education - secondary	0.638	+0.003
Pre-delivery			
1	Antepartum hemorrhage	0.558	NA
2	Cluster perinatal mortality	0.644	+0.086
3	Gestational age at enrollment	0.642	-0.002
4	Hypertension/severe pre-eclampsia/eclampsia	0.660	+0.018
5	Maternal age	0.662	+0.002
6	Birth order	0.668	+0.006
7	Parity	0.677	+0.009
8	Delivery by traditional birth attendant	0.686	+0.009
9	Delivery by physician	0.697	+0.011
10	Maternal education – none	0.702	+0.005
11	Delivery by nurse/nurse midwife/lady health worker/health worker	0.723	+0.021
12	Clinic/health center delivery	0.731	+0.029
13	Hospital delivery	0.731	0
14	≥1 prenatal visit	0.736	+0.005
15	Maternal education – primary	0.735	-0.001

*Predictors are added consecutively using the Random Forest model; then AUC calculated.

Hypertensive disease/severe pre-eclampsia/eclampsia defined as blood pressure >140/90 mm Hg, proteinuria, and seizures. The order used in this table was the order of importance assigned by the predictive model assessment of important predictors.

© 2020 Shukla VV et al. *JAMA Network Open.*

eTable3: Risk Score Model Coefficients and Calculation (Neonatal Mortality Outcome)

Delivery/day-1				
	Coefficient	Standard Err	95% Confidence Interval	Scoring Weights
Intercept	-4.8569	0.203	(-5.254, -4.46)	
Birth order	0.0467	0.109	(-0.167, 0.261)	0.5
Maternal age <20 years	-0.197	0.147	(-0.486, 0.091)	-2.0
Maternal age 20-35 years	-0.2624	0.113	(-0.483, -0.042)	-2.6
≥1 prenatal visit	-0.2817	0.082	(-0.443, -0.12)	-2.8
Delivery by physician	-0.1161	0.077	(-0.266, 0.034)	-1.2
Delivery by nurse/nurse midwife/lady health worker/health worker	-0.3855	0.076	(-0.534, -0.237)	-3.9
Delivery by traditional birth attendant	-0.0742	0.076	(-0.224, 0.075)	-0.7
Birthweight by gram*[1]	-0.7493	0.021	(-0.79, -0.708)	-7.5
Bag and mask resuscitation	2.0383	0.052	(1.937, 2.14)	20.4
Gestational age by week at enrollment*[2]	0.1	0.022	(0.057, 0.143)	1.0
Parity*[3]	0.1024	0.044	(0.016, 0.189)	1.0
Vaginal delivery	-0.063	0.054	(-0.169, 0.043)	-0.6
Vaginal assisted delivery	0.2307	0.141	(-0.046, 0.507)	2.3
Obstructed labor	-0.154	0.08	(-0.31, 0.002)	-1.5
Gestational age by week*[4]	-0.1893	0.016	(-0.22, -0.159)	-1.9
Cluster perinatal mortality	4.4816	0.915	(2.689, 6.274)	44.8
Antepartum hemorrhage	0.3526	0.095	(0.167, 0.538)	3.5
Multiple birth	-0.1159	0.086	(-0.284, 0.053)	-1.2
Hypertension/severe pre-eclampsia/eclampsia	0.1057	0.069	(-0.03, 0.241)	1.1
Antenatal corticosteroids	-0.0129	0.071	(-0.152, 0.126)	-0.1
Suspected maternal sepsis	0.6125	0.165	(0.29, 0.935)	6.1
Male sex	0.2495	0.032	(0.186, 0.313)	2.5
Gestational age by week at enrollment squared*[5]	-0.0312	0.017	(-0.064, 0.002)	-0.3
Gestational age by week at enrollment* times birthweight*[6]	0.0176	0.014	(-0.01, 0.045)	0.2
Parity* times mother 20- 35 years at enrollment[7]	0.0232	0.047	(-0.069, 0.115)	0.2
Parity* times birthweight by gram*[8]	0.0239	0.011	(0.003, 0.045)	0.2
Vaginal delivery times obstructed labor[9]	0.6638	0.106	(0.457, 0.871)	6.6
Birthweight by gram* squared[10]	0.1425	0.007	(0.129, 0.156)	1.4
Birthweight by gram* times bag and mask resuscitation[11]	0.4209	0.028	(0.366, 0.476)	4.2
Post-delivery/day-2				
	Coefficient	Std Err	95% CI	Scoring Weights
Intercept	-5.7805	0.2	(-6.172, -5.389)	
Birth order	0.0595	0.121	(-0.177, 0.296)	0.6
Maternal age <20 years	-0.0788	0.106	(-0.286, 0.128)	-0.8

© 2020 Shukla VV et al. *JAMA Network Open.*

Maternal age 20-35 years	-0.0789	0.086	(-0.247, 0.089)	-0.8
Maternal education- none	0.2402	0.098	(0.048, 0.432)	2.4
Maternal education- primary	0.3518	0.09	(0.175, 0.529)	3.5
Maternal education- secondary	0.2334	0.087	(0.062, 0.404)	2.3
≥1 prenatal visit	-0.5184	0.089	(-0.694, -0.343)	-5.2
Birthweight by gram*[1]	-0.8003	0.024	(-0.848, -0.753)	-8.0
Bag and mask resuscitation	0.9115	0.052	(0.81, 1.013)	9.1
Neonatal hospitalization	2.4699	0.064	(2.345, 2.595)	24.7
Gestational age by week at enrollment*[2]	0.0958	0.021	(0.055, 0.136)	1.0
Gestational age by week*[4]	-0.1079	0.022	(-0.151, -0.064)	-1.1
Neonatal antibiotics	1.4685	0.068	(1.336, 1.601)	14.7
Medicinal cord care	0.3106	0.065	(0.183, 0.439)	3.1
Cluster perinatal mortality	8.2783	1.229	(5.869, 10.688)	82.8
Parity*[3]	0.0974	0.021	(0.057, 0.138)	1.0
Multiple birth	-0.0381	0.095	(-0.224, 0.148)	-0.4
Antenatal corticosteroids	-0.0655	0.081	(-0.225, 0.094)	-0.7
Hospital delivery	-0.0292	0.05	(-0.127, 0.069)	-0.3
Maternal antibiotics	-0.0702	0.046	(-0.161, 0.02)	-0.7
Delivery by physician	-0.0104	0.052	(-0.113, 0.092)	-0.1
Gestational age by week at enrollment* squared[5]	-0.0532	0.019	(-0.091, -0.016)	-0.5
Maternal age <20 years times neonatal hospitalization[12]	1.0243	0.1	(0.828, 1.22)	10.2
Gestational age by week* squared[13]	0.028	0.006	(0.016, 0.04)	0.3
Birthweight by gram* squared[10]	0.1196	0.007	(0.106, 0.133)	1.2
Birthweight by gram* times neonatal hospitalization[14]	0.4362	0.032	(0.374, 0.499)	4.4
Birthweight by gram* times neonatal antibiotics[15]	0.436	0.031	(0.375, 0.497)	4.4
Neonatal antibiotics times medicinal cord care[16]	0.3047	0.106	(0.096, 0.513)	3.0

Hypertensive disease/severe pre-eclampsia/eclampsia defined as blood pressure >140/90 mm Hg, proteinuria, and seizures;

Suspected maternal sepsis defined as fever with pelvic pain and abnormal vaginal discharge (foul-smelling or presence of pus);

Medicinal cord care: application of chlorhexidine or any other medicine to the umbilical cord;

*=standardized

Standardization of the variables:

© 2020 Shukla VV et al. *JAMA Network Open.*

[1] Birthweight must be standardized by subtracting 2,898.103 and then dividing by 505.0561.

[2] Gestational age at enrollment must be standardized by subtracting 20.159 and then dividing by 9.2468.

[3] Parity must be standardized by subtracting 1.788 and then dividing by 2.1019.

[4] Gestational age at delivery must be standardized by subtracting 38.556 and then dividing by 3.5519.

[5] Gestational age at enrollment must be standardized and then squared.

[6] Gestational age at enrollment must be standardized, and birthweight must be standardized, then the two must be multiplied.

[7] Parity must be standardized and then multiplied by the indicator for mother's age between 20-35 years at enrollment.

[8] Parity and birthweight must be standardized, then the two must be multiplied.

[9] The indicator for vaginal delivery must be multiplied by the indicator for the mother having obstructed/prolonged labor/failure to progress

[10] Birthweight must be standardized, then squared.

[11] Birthweight must be standardized and then multiplied by the indicator for bag and mask resuscitation.

[12] The indicator for the mother being <20 years old at enrollment must be multiplied by the indicator for the neonate having at least one condition requiring hospitalization.

[13] Gestational age at delivery must be standardized and then squared.

[14] Birthweight must be standardized and then multiplied by the indicator for the neonate having at least one condition requiring hospitalization.

© 2020 Shukla VV et al. *JAMA Network Open.*

[15] Birthweight must be standardized and then multiplied by the indicator for the neonate having received antibiotics.

[16] The indicator for the neonate having received antibiotics must be multiplied by the indicator for medicinal cord care.

Once standardized, the variables need to be multiplied by the scoring weight to get the contribution of the variable to the overall risk.

Negative weights represent risk factors or interaction whose presence results in a reduction in mortality risk when the risk factor or product of risk factors increase, while positive weights represent risk factors or interactions whose presence increases the mortality risk when the risk factor or product of risk factors increase. The total risk score is the sum of all products between risk score weights and corresponding sample data. Once all variables have been multiplied by the weights, the products need to be summed to calculate the total risk score.

© 2020 Shukla VV et al. *JAMA Network Open.*

eTable4: Mortality probability functions for the total risk score (Neonatal Mortality Outcome)

Scenario	Outcome probability as a function of Total Risk Score	Validation AUC
Delivery/day-1	$\frac{\exp(-4.9647 + 0.1003 * \text{RiskScore})}{1 + \exp(-4.9647 + 0.1003 * \text{RiskScore})}$	0.809
Post-delivery/day-2	$\frac{\exp(-5.8822 + 0.1000 * \text{RiskScore})}{1 + \exp(-5.8822 + 0.1000 * \text{RiskScore})}$	0.845

Risk score calculation as explained in eTable3. AUC=Area under the curve.

© 2020 Shukla VV et al. *JAMA Network Open.*

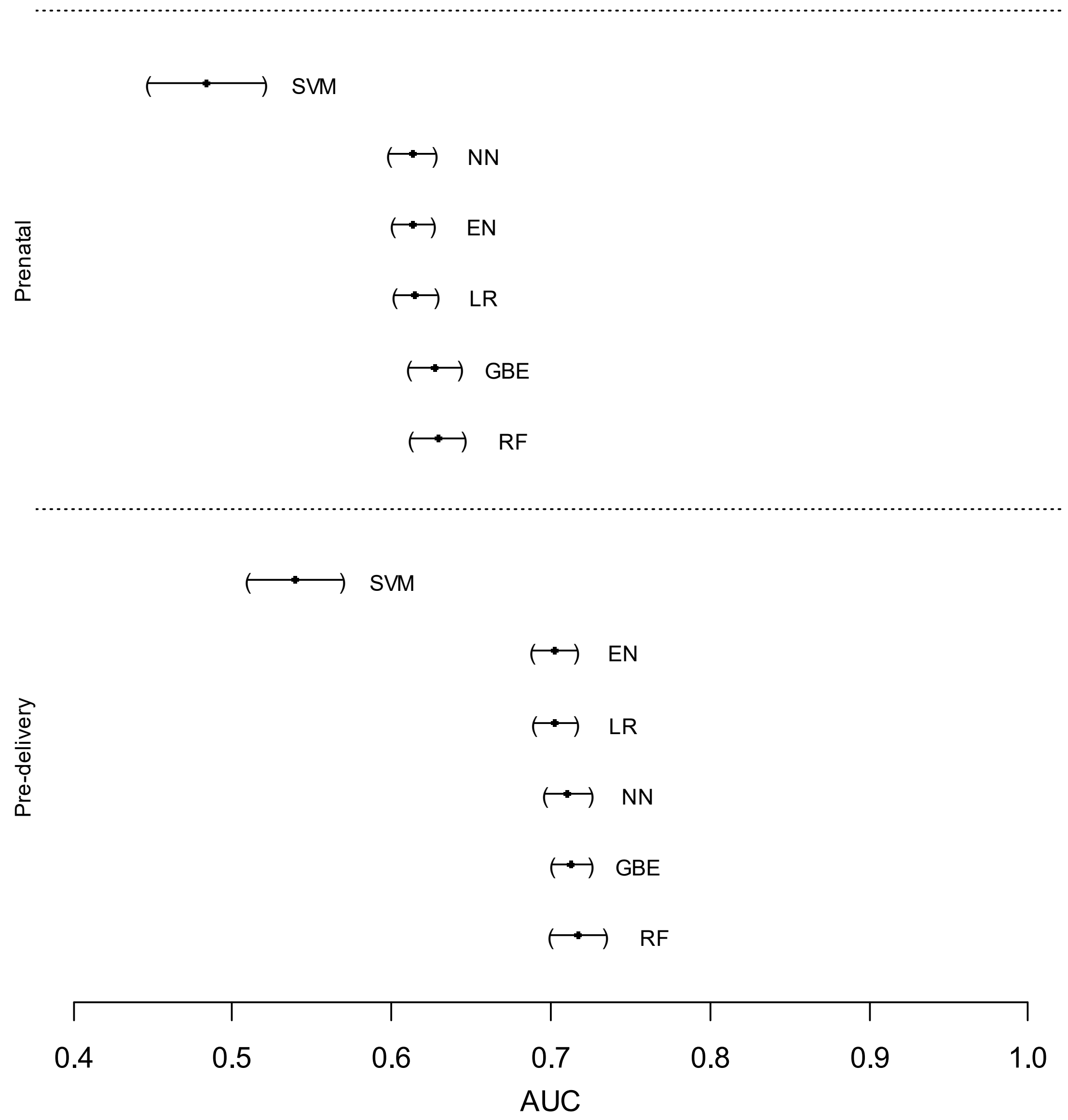

eFigure1: Mean (95%CI) for validation AUC by scenario for outcomes of fresh stillbirth.

The analysis of models for fresh stillbirth showed that all models based on prenatal variables had AUC values ≤0.63 and pre-delivery models had AUC values ≤0.72. SVM=support vector machine with radial basis function kernel, EN=logistic elastic net, LR=logistic regression, NN=neural network, GBE=gradient boosted ensemble, and RF=random forest, CI=Confidence Interval, AUC=area under the curve.

© 2020 Shukla VV et al. *JAMA Network Open.*

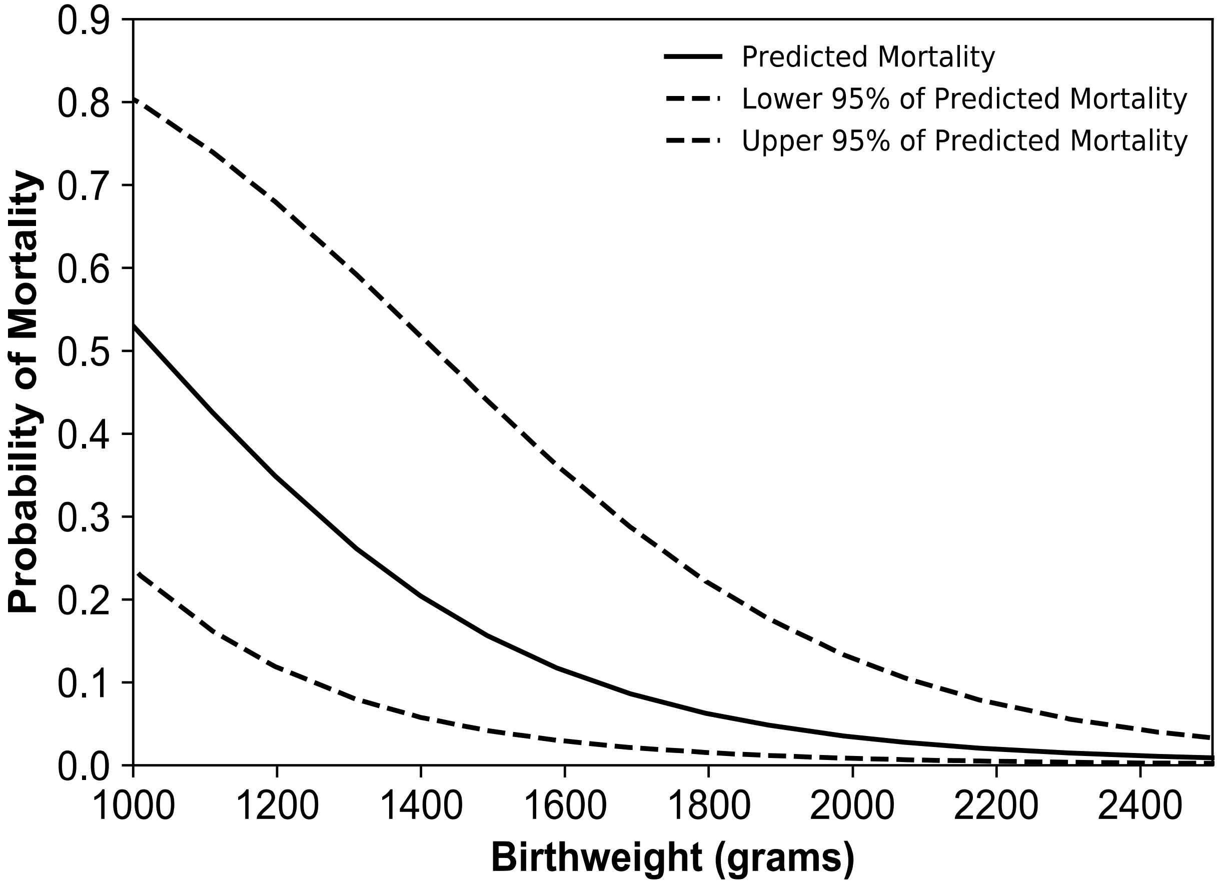

eFigure2: Probability of mortality as a function of birthweight, post-delivery/day-2 scenario. Graph derived from mortality predictions taken from the post-delivery/day-2 logistic regression model presented in eTable2. Predictions from the logistic regression model were binned into groups of 100-gram windows centered at 525, 800, 900, 1000, 1100, … 4200, 4300, and 5675 grams and then averages and standard deviations (SD) calculated within each bin. Using the bin averages and standard deviations, lower and upper limits were calculated using average +/- 1.96*SD. Graphing was further enhanced by fitting cubic splines to the bin averages and lower/upper limit calculations as well as reducing the plot to the range of 1000 to 2400 grams. The process of binning and smoothing the averages and confidence interval line was used to

© 2020 Shukla VV et al. *JAMA Network Open.*

graphically illustrate the relationship between birthweight probability of mortality while averaging over the other predictors.

eREFERENCES

1. Ambalavanan N, Carlo WA, Bobashev G, et al. Prediction of death for extremely low birth weight neonates. *Pediatrics.* 2005;116(6):1367-1373.
2. Eggleston B, Dismuke-Greer CE, Pogoda TK, et al. A prediction model of military combat and training exposures on VA service-connected disability: a CENC study. *Brain inj.* 2019:1-13.
3. SAS Institute Inc (2002-2012). SAS/STAT Software, Version 9.4. Cary. http://www.sas.com/. Accessed 2020-09-01.
4. Pedregosa F, Varoquaux G, Gramfort A, et al. Scikit-learn: Machine learning in Python. *J Mach Learn Res.* 2011;12:2825-2830.
5. R Core Team (2020). R: A language and environment for statistical computing. R Foundation for Statistical Computing, Vienna, Austria. https://www.R-project.org/. Accessed 2020-09-01.
6. Hanley JA, McNeil BJ. The meaning and use of the area under a receiver operating characteristic (ROC) curve. *Radiology.* 1982;143(1):29-36.
7. Nembrini S, Konig IR, Wright MN. The revival of the Gini importance? *Bioinformatics.* 2018;34(21):3711-3718.
8. van der Ploeg T, Austin PC, Steyerberg EW. Modern modelling techniques are data hungry: a simulation study for predicting dichotomous endpoints. *BMC Med Res Methodol.* 2014;14:137.

© 2020 Shukla VV et al. *JAMA Network Open.*
